# Accelerating scientific discoveries through data-driven innovations

**DOI:** 10.1016/j.patter.2023.100876

**Published:** 2023-11-10

**Authors:** Francis J. Alexander, Meifeng Lin, Xiaoning Qian, Byung-Jun Yoon

**Affiliations:** Computing, Environment and Life Sciences, Argonne National Laboratory; Computational Science Initiative, Brookhaven National Laboratory; Department of Electrical and Computer Engineering, Texas A&M University; Computational Science Initiative, Brookhaven National Laboratory; Department of Electrical and Computer Engineering, Texas A&M University; Computational Science Initiative, Brookhaven National Laboratory

Developing artificial intelligence (AI) and machine learning (ML) methods that can accelerate scientific discoveries and advance science has become one of the important research directions for the AI/ML research community. It has been gaining increasing attention from researchers in diverse scientific areas, including biomedical science, materials science, climate science, physics, chemistry, and many others. Data-driven AI/ML innovations to enable reliable predictions and optimal decision making for scientific discoveries face several critical challenges, among which are high system complexity, large search space, incomplete knowledge, and small data, all of which demand novel strategies to effectively address them. Meeting these challenges and thereby accelerating scientific discoveries and industrial innovations, calls for research that can take full advantage of the latest advances in AI/ML to integrate data-driven techniques with scientific knowledge and is able to execute them in modern high-performance computing (HPC) environments at scale. This *Patterns* special collection “Accelerating scientific discoveries through data-driven innovations” features articles that showcase the promising roles of AI/ML and data-driven modeling in accelerating scientific discoveries and may inspire the next wave of data-driven innovations in various scientific domains.

AI/ML models are becoming increasingly popular in materials science research as they enable computationally efficient prediction of material properties and thereby can facilitate the discovery of novel materials with desirable properties. Effective property prediction using AI/ML models requires accurate structural information of the material, which is unavailable for new materials. However, *ab initio* prediction of equilibrium structures is computationally expensive, creating a significant bottleneck in materials discovery pipelines. In the paper “Strain data augmentation enables machine learning of inorganic crystal geometry optimization,” Dinic et al. address this challenge by training an ML model that can predict the impact of local and global strains and enhance energy predictions for structural deformations. The trained model allows efficient ML-based geometry optimization that leads to improved prediction of formation energy for inorganic crystals.

A common challenge in leveraging AI/ML in scientific applications is the scarcity of relevant data that can be used to robustly train accurate data-driven models. One potential approach for alleviating this issue is domain adaptation (DA), which refers to a technique that aims to improve the performance of a model in a “target” domain of interest that is different from the “source” domain in which the model was originally trained. Typically, DA techniques can be helpful for training an ML model in a data-poor “target” domain by leveraging the data in relevant “source” domain(s) that may be relatively data rich. The work by Wu et al. proposes an optimal transport method, called MROT, that enables unsupervised and semi-supervised DA for molecular regression tasks to facilitate the exploration of new molecules in small data regimes.

While the development of novel AI/ML techniques is rapidly transforming scientific research, there is no doubt that such transformation brought forth by rapid advances in AI/ML would not have been possible without advances in modern HPC. Motivated by the fact that the semiconductor industry is getting closer to the limit of chip miniaturization, Weidner et al. explore quantum computing as an alternative paradigm for simulating complex systems. Based on gene regulatory networks modeled by Boolean logic, the authors demonstrate how a quantum algorithm using qubits can effectively analyze the network dynamics despite the exponential growth of network states and the simulation complexity.

An important application area for AI/ML that is receiving growing attention in science and engineering is the utilization of advanced AI/ML techniques for real-time monitoring, safe and efficient management, and autonomous control of complex devices, equipment, or facilities. In the paper entitled “Long-sequence voltage series forecasting for internal short circuit early detection of lithium-ion batteries,” Cui et al. showcase the use of AI/ML for early detection of internal short circuits (ISCs), a common safety concern for lithium-ion batteries. Using a deep learning scheme based on an encoder-decoder architecture, the method is capable of predicting long-sequence voltage and power series where the evolution inconsistency between normal and ISC battery voltage can be used for accurate ISC detection. Similarly, one may envision the use of AI/ML for continuously monitoring the health, safety, and efficiency of complex systems—such as wind turbines or power grids—as well as the autonomous control of experimental facilities—such as complex beamlines and associated detectors—for scientific experiments.

In recent years, data-driven methods for molecular optimization have received increasing attention—especially for applications such as drug discovery and materials design—because of their potential to significantly accelerate the discovery of novel molecules with desired properties. Devising effective methods for multi-objective optimization is critical in molecular design, as one typically needs design molecules that satisfy multiple—and often competing—design criteria. In the review article entitled “Computer-aided multi-objective optimization in small molecule discovery,” Fromer and Coley focus on Pareto optimization algorithms for multi-objective molecular design. Although it is commonplace, mainly for simplicity, to combine multiple properties via scalarization to define a single objective function, Pareto optimization methods have important advantages as they obviate the need to pre-determine the relative importance between different properties and as they can reveal the trade-offs between competing properties. In addition to providing a comprehensive review of both pool-based and *de novo* generative approaches for multi-objective molecular design, the authors also discuss open problems and opportunities for future research.

The review paper by Qian et al. entitled “Knowledge-driven learning, optimization, and experimental design under uncertainty for materials discovery” discusses several salient challenges in applying data-driven methods to real-world complex systems and presents recent advances in the field for addressing them. The central issue is the complexity of real-world systems for which existing data may be insufficient for accurate system modeling or identification. Moreover, acquisition of ample data may be practically difficult or formidably expensive, as a result of which existing ML methods may fail to produce reliable and generalizable predictions. Qian et al. show how uncertainty-aware and objective-driven Bayesian signal processing and machine learning schemes can enable effective optimization and experimental design for complex uncertain systems. Furthermore, this review article showcases how knowledge-driven and physics-informed approaches can be critical for effective learning in data-limited scientific domains.

High-performance computing has been playing central roles in various scientific domains, and its importance has been growing even further as a result of the increasing adoption of AI/ML models in scientific inquiries. Executing large-scale computational campaigns requires decision making to efficiently allocate the computational resources to ensure that we obtain the desired outcomes while minimizing the execution time or computational cost that is incurred. Traditionally, such decision making has relied on the intuition and experience of domain experts, which may yield reasonably good but suboptimal results. In the paper “Optimal decision-making in high-throughput virtual screening pipelines,” Woo et al. tackle this optimal computational campaign (OCC) design problem in the context of high-throughput virtual screening (HTVS) campaigns. This work shows that by optimizing the operational policy (i.e., the screening policy in this study), one can substantially accelerate screening virtually without any degradation in the screening accuracy.

Although the papers included in this *Patterns* special collection showcase some of the fascinating ways that data-driven innovations can accelerate scientific discoveries, they are by no means comprehensive. We expect that we will see continued significant data-driven discoveries and rapid advances across diverse scientific domains in the upcoming years through cross-cutting research at the interactions of AI/ML, mathematics, and computing.

